# A coupled microscopy approach to assess the nano-landscape of weathering

**DOI:** 10.1038/s41598-019-41357-0

**Published:** 2019-03-29

**Authors:** Rebecca A. Lybrand, Jason C. Austin, Jennifer Fedenko, Rachel E. Gallery, Erin Rooney, Paul A. Schroeder, Dragos G. Zaharescu, Odeta Qafoku

**Affiliations:** 1 Department of Crop and Soil Science, Oregon State University, Corvallis, OR 97331 USA; 2 Department of Geology, University of Georgia, Athens, GA 30602 USA; 3 School of Natural Resources and the Environment, University of Arizona, Tucson, AZ 85721 USA; 4 Department of Ecology and Evolutionary Biology, University of Arizona, Tucson, AZ 85721 USA; 5 School of Earth and Atmospheric Sciences, Georgia Institute of Technology, Atlanta, GA 30332 USA; 6 Pacific Northwest National Laboratory, Richland, WA 99352 USA

## Abstract

Mineral weathering is a balanced interplay among physical, chemical, and biological processes. Fundamental knowledge gaps exist in characterizing the biogeochemical mechanisms that transform microbe-mineral interfaces at submicron scales, particularly in complex field systems. Our objective was to develop methods targeting the nanoscale by using high-resolution microscopy to assess biological and geochemical drivers of weathering in natural settings. Basalt, granite, and quartz (53–250 µm) were deployed in surface soils (10 cm) of three ecosystems (semiarid, subhumid, humid) for one year. We successfully developed a reference grid method to analyze individual grains using: (1) helium ion microscopy to capture micron to sub-nanometer imagery of mineral-organic interactions; and (2) scanning electron microscopy to quantify elemental distribution on the same surfaces via element mapping and point analyses. We detected locations of biomechanical weathering, secondary mineral precipitation, biofilm formation, and grain coatings across the three contrasting climates. To our knowledge, this is the first time these coupled microscopy techniques were applied in the earth and ecosystem sciences to assess microbe-mineral interfaces and *in situ* biological contributors to incipient weathering.

## Introduction

The weathering of Ca- and Mg-silicate rocks stabilizes global climate over geologic timescales by consuming carbon dioxide (CO_2_) as carbonic acid and nourishes the biosphere by supplying rock-derived nutrients to microorganisms and plants^1–3^. Microorganisms represent the bulk of subsurface terrestrial biomass, colonize almost all terrestrial environments, and are an important component in the biogeochemical cycling of carbon, nitrogen, and other life-supporting elements^4^. Microorganisms also actively and selectively interact with mineral surfaces by engaging in element capture and transfer processes from ecosystem to molecular levels. This is best demonstrated by the mycorrhiza “underground highway” that facilitates the transfer of carbon and nutrients within and between plants through a common hyphal network^5,6^. The feedback loops that plants and their rhizosphere microbiome develop with bedrock minerals emerged in the early Phanerozoic (as early as Devonian, 407 million years ago) during the colonization of terrestrial habitats by plants^7–9^, and continues to evolve in the present. However, knowledge gaps remain in characterizing biological-mineral interfaces at fundamental scales of interactions that dictate how the abiotic and biotic components of ecosystems contribute to elemental cycles and ecosystem function^10,11^. The study of biogeochemical interactions in soil can be aided by novel technologies that can enhance our ability to assess mineral surfaces and associated organisms at micro- to nanoscale resolutions. We demonstrate potential applications of Helium Ion Microscopy (HIM) as an emerging tool in the earth and ecological sciences in combination with Scanning Electron Microscopy (SEM)-Energy Dispersive X-ray spectra (EDX) using, as an example, samples from a field study.

Helium ion microscopy is a relatively new technology mostly used in the material and biomedical sciences that is well-suited for research in the natural sciences^12,13^. HIM has become renowned for its sensitivity for imaging surfaces and interfaces; its high-resolution imaging capabilities with applications in the sub-nanometer range for secondary electron imaging; and for the ability to image biological specimens with little to minimal damage to the sample given the low mass of He ions compared to other ion sources^14^. The low excitation volume of the helium ion beam allows for sharper, higher resolution images of surface materials to be obtained compared to SEM^15^. Helium ion microscopy offers an opportunity to assess natural nanomaterials produced through biogeochemical processes^16^, and more specifically, provides an avenue for addressing how microorganisms (primarily bacteria and fungi; collectively referred to herein as microbes) transform minerals at submicron scales.

Unresolved questions pertaining to mineral transformation mechanisms involve assessing relative controls of biochemical versus biomechanical weathering, indirect or direct microbe-mineral interactions, and whether microbes enhance or slow mineral dissolution^17,18^. Microscopy studies have examined biomechanical and biochemical weathering mechanisms that: weaken mineral structures through the fungal-driven oxidation of Fe(II) in biotite^19^, induce secondary mineral formation and biomineralization^20^, enhance microbial growth around nutrient-rich zones^21^, incongruently leach major and trace elements^10,22^, and exert control of K nutrient uptake and related clay mineral modifications under changing land-use regimes^23^. Atomic force microscopy has been used to detect the simultaneous occurrence of indirect and direct biochemical weathering, where it was observed that abundant small etching pits formed on mineral surfaces exposed to siderophores (molecular Fe chelators) released to solution by microbes, whereas fewer yet larger “biopits” formed on surfaces colonized directly by bacteria^24^. Fungal biofilms imaged with SEM were shown to enhance biochemical weathering through the release of organic acids at the hypha-mineral interface and transformed grains via shrink-swell processes^25^. Backscattered electron images captured by SEM were utilized to characterize polysaccharide biofilms produced by microbes that also affect weathering by promoting mineral dissolution or serving as a protective coating that slows weathering^26,27^. Biochemical processes may have a larger effect on weathering than biomechanical transformations^18^ as observed in saturated liquid cultures where fungi accelerated biotite dissolution by acidifying the bulk solution at greater rates than direct fungal-mineral interactions^28^, but this may not be the case in unsaturated conditions found in field settings. High resolution imaging and elemental mapping presents an opportunity to target biological weathering processes occurring at micron to submicron scales^19,29,30^. Coupling the superior imaging capabilities of HIM with established methods for measuring elemental distribution using SEM can help resolve complex weathering processes found in natural soil environments.

Bacteria and fungi intricately interact with silicate minerals to promote weathering; however, it is not completely understood how surface morphology and elemental distribution in mineral grains (a fundamental weathering scale unit) change under interactions with abiotic and biotic factors operating at various scales. In-soil mesh bags represent a successful approach to assess the response of soil fungal communities to contrasting soil nutrient patches^31^ or to deployed mineral substrates that vary in nutrient content^32–35^. In-soil mesh bags have also been used to estimate fungal biomass in the field^36^, to explore the role of mycorrhizal fungi as soil carbon sinks^37^, and to examine how forest soil nutrient status impacts soil mycorrhizal foraging activities^38^. Mesh bags filled with granular rock substrates also showed how mycorrhizal fungi employ “biosensing” mechanisms to preferentially colonize and weather basalt compared to granite and quartz under both laboratory conditions^39^ and in forest systems^40^.

Our objective was to employ HIM and SEM to detect potential drivers and evidence of mineral weathering at the micron-submicron scale using in-soil mesh bags filled with granular substrates that were deployed in natural field settings. We hypothesized that a reference locator grid would be effective to identify and analyze individual grains by applying: (1) surface sensitive helium ion microscopy to capture details at micron to sub-micrometer scales and to image mineral-organic matter interaction hotspots on grain surfaces; and (2) scanning electron microscopy to quantify elemental distribution at the same locations using electron backscattering for chemical point analyses and elemental maps. We used a field experiment to demonstrate the effectiveness of our microscopy approach for assessing incipient weathering of grains exposed to the natural environment. Briefly, nylon mesh bags filled with granular granite, basalt, and quartz (53–250 µm) were retrieved from surface soils (0–10 cm) of contrasting climates (semiarid, subhumid, and humid) and landscape positions (convergent footslope, divergent summit) after one year of deployment. The substrates were sealed into coarse (35 µm mesh size) and fine (0.5 µm mesh size) nylon mesh bags designed to include and exclude direct fungal hyphae contact with mineral surfaces. Both mesh sizes allow for solute, nanophase, and bacterial interactions with mineral grains. To our knowledge, this is the first time HIM has been used in conjunction with SEM to characterize complex field samples in the earth and ecosystem sciences, and we provide a benchmark method to assess microbe-mineral interfaces and biological inputs to incipient weathering in field and lab experiments at micro- to nanometer resolutions.

## Results and Discussion

### Helium ion microscopy

**Figure 1:**
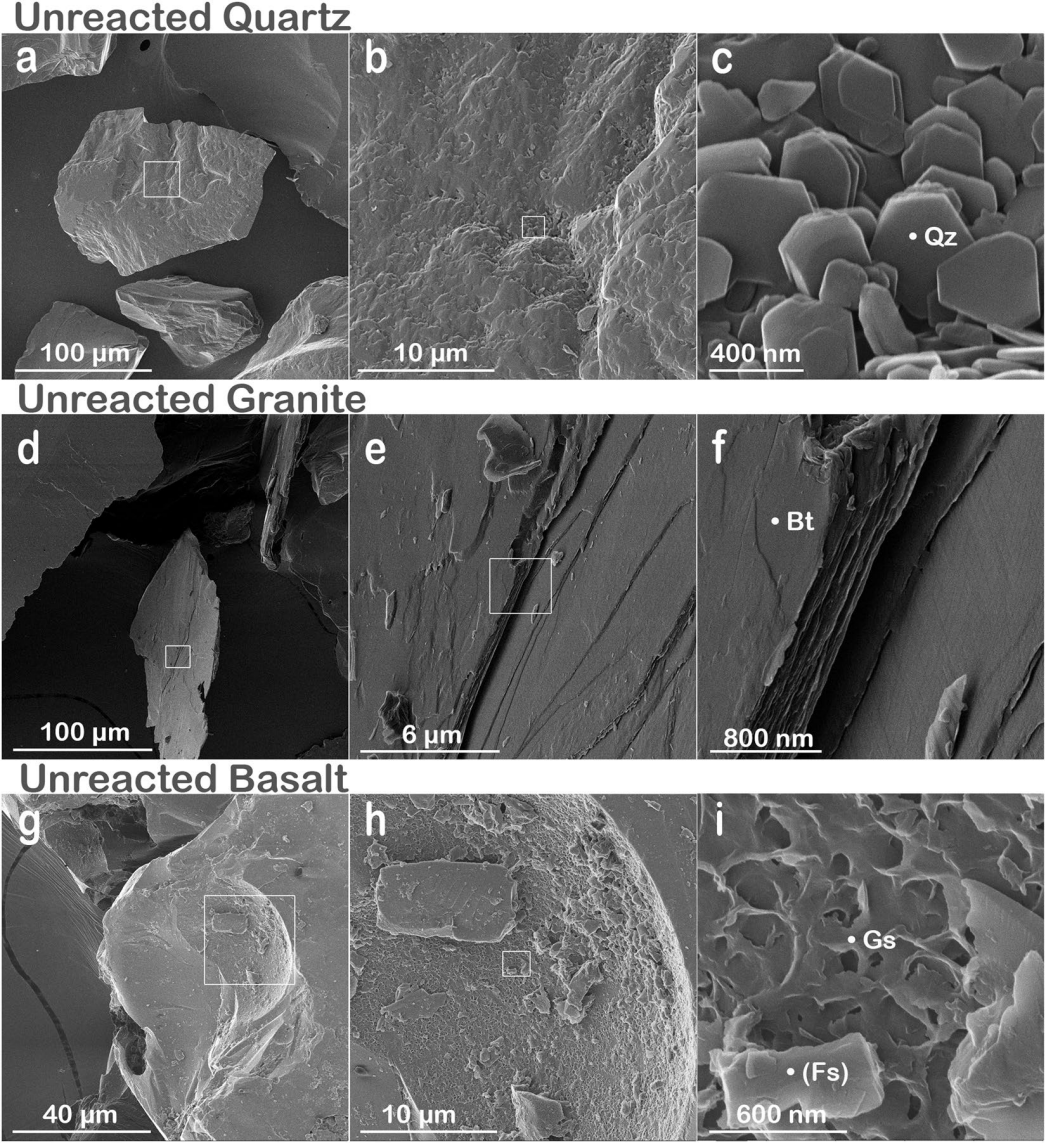
Undeployed granular rocks. Helium ion micrographs showing the diversity of surface topography of the three rocks including **(a–c)** hexagonal crystals in quartz (Qz), **(d**–**f)** the lamellar structure of biotite (Bt) in granite, and **(g–i)** the trabecular surface of the basaltic glass matrix (Gs) with a potential feldspar [(Fs)] embedded. In each case, the nanoscale images highlight within each sample a diversity of mineral surfaces. The different lattice planes and dimensions offer a variety of nano-landscapes for mineral weathering, which can both enhance or inhibit biomechanical and biochemical transformations induced by microbes. Undeployed (control) granular substrates were imaged for comparison with field-deployed samples.

The high-resolution imaging of grain surfaces and microbe-mineral interfaces revealed the intricacies of such features in unprecedented detail. We first examined untreated granular substrate controls and distinguished grain microtopography and nanocrystal edges characteristic of quartz substrate (Fig. 1a–c), the lamellar structures and smooth surfaces of biotite monominerals in granite (Fig. 1d–f), and basalt vesicles embedded with amorphous glass, with trabecular inner surface topography (Figs 1g–i; S1, S2, S3). These surfaces are expected to respond differently to similar physical, chemical, and biological agents during weathering, and represent a source of chemical entities that subsequently feed into global biogeochemical cycles.

**Figure 2:**
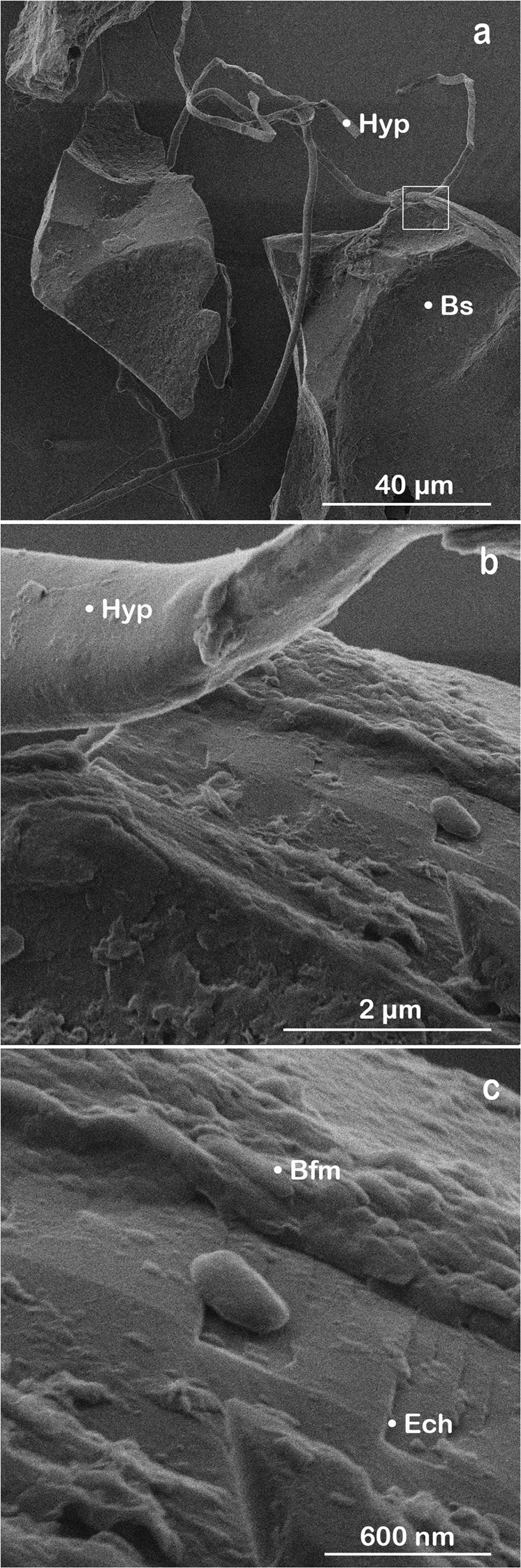
Helium ion micrographs of a fungal-mineral interface. Basalt grains (Bs; 53–250 µm) exposed to biological interactions at 10 cm soil depth in the semiarid climate (Catalina desert scrub ecosystem, S. Arizona) for one year. **(a)** The imaging shows fungal hyphae (Hyp) connecting mineral grains and **(b)** adhering to mineral surfaces. **(c)** The fungal-mineral contact area shows its footprint in the biofilm (Bfm) and nanoscale evidence of mineral etching (Ech).

Helium ion microscopy further revealed interactions among fungal hyphae networks with the granular granite, basalt, and quartz in the coarse mesh bags (35 µm mesh size) in all ecosystems, i.e., semiarid Catalina desert scrub, subhumid Catalina mixed conifer forest, and humid Calhoun mixed hardwood-pine forest. For instance, basalt-fungal complexes in the Catalina desert ecosystem displayed morphological variability across resolutions (Fig. 2): micron scale imaging revealed hyphae connecting mineral grains (Fig. 2a) as well as growing along grain edges and surfaces (Fig. 2b). Nanoscale imaging of the same interface suggested accelerated weathering of the mineral surface due to biological interactions, as indicated by a contrast in grain texture between a site on the mineral surface previously in contact with a fungal hypha versus an adjacent smooth site with no evidence of hypha interaction (Fig. 2c). Likewise, nanoscale mineral coatings are visible on each side of the fungal-mineral contact (Fig. 2c).

Our results demonstrate that HIM is well-suited for the non-quantitative imaging of microbe-mineral interfaces that are common to the environmental and ecosystem sciences. Specifically, we present potential evidence for incipient microbial-mineral interactions, biological specimens in the weathering environment with contrasting morphological characteristics, and weathering features on grains at different resolutions. High resolution imaging with HIM has been utilized in the nanosciences^12–14,41–43^ and biomedical research^44,45^. However, HIM is often overlooked as a visualization tool to resolve complex environmental samples where various abiotic and biotic actors are at play, despite its elevated sensitivity and higher resolution capabilities compared to classical electron microscopy techniques.

### Biomechanical and biochemical weathering

**Figure 3:**
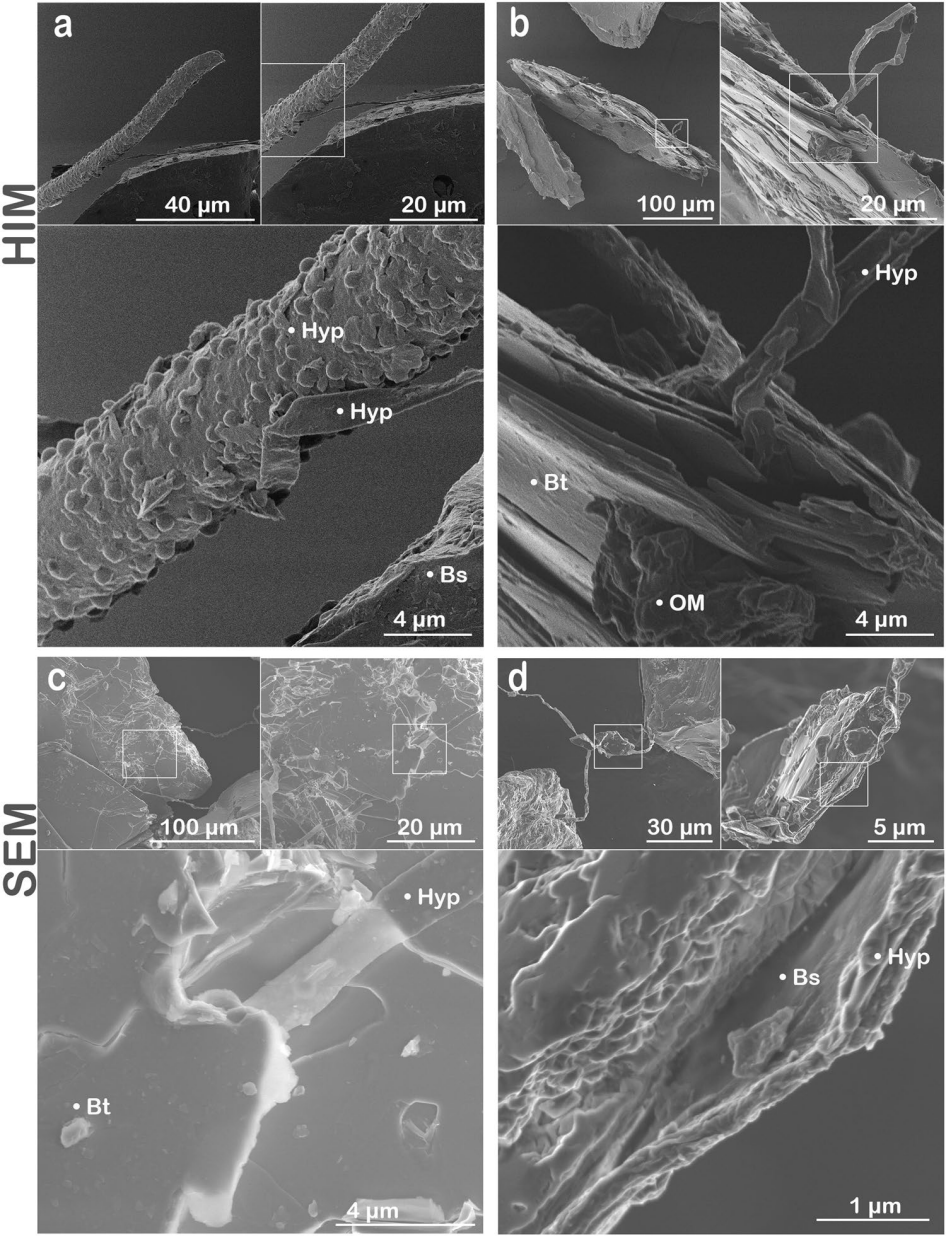
Types of biomechanical activity. HIM (**a** and **b**) and SEM (**c** and **d**) imagery showing examples for biological inputs and biomechanical weathering in granular samples deployed in the subhumid (**a–c**; Catalina mixed conifer forest) and humid (**d**; Calhoun mixed hardwood-pine forest) field sites. Examples include **(a)** distinct fungal structural units with hyphal (Hyp) growth along the edges of a basaltic (Bs) grain where fungi exhibited unique surface morphologies at microscale resolutions. **(b)** Fungal hyphae interacting with biotite (Bt) mica from granite samples including hyphal growth between the interlayer sheets of biotite along the grain edge that represents a form of biomechanical weathering. **(c)** A fungal hypha (Hyp) also penetrated a surface layer sheet of biotite (Bt) as observed in secondary electron images. (**d**) Fungal-mineral interactions were also imaged for the Calhoun mixed hardwood-pine forest including a hypha adhered across and along the edge of a basaltic grain. The fungal-mineral interface provided potential evidence for hyphal tunneling or an abiotic weathering feature enhanced by contact with the fungal hypha along the grain edge.

**Figure 4:**
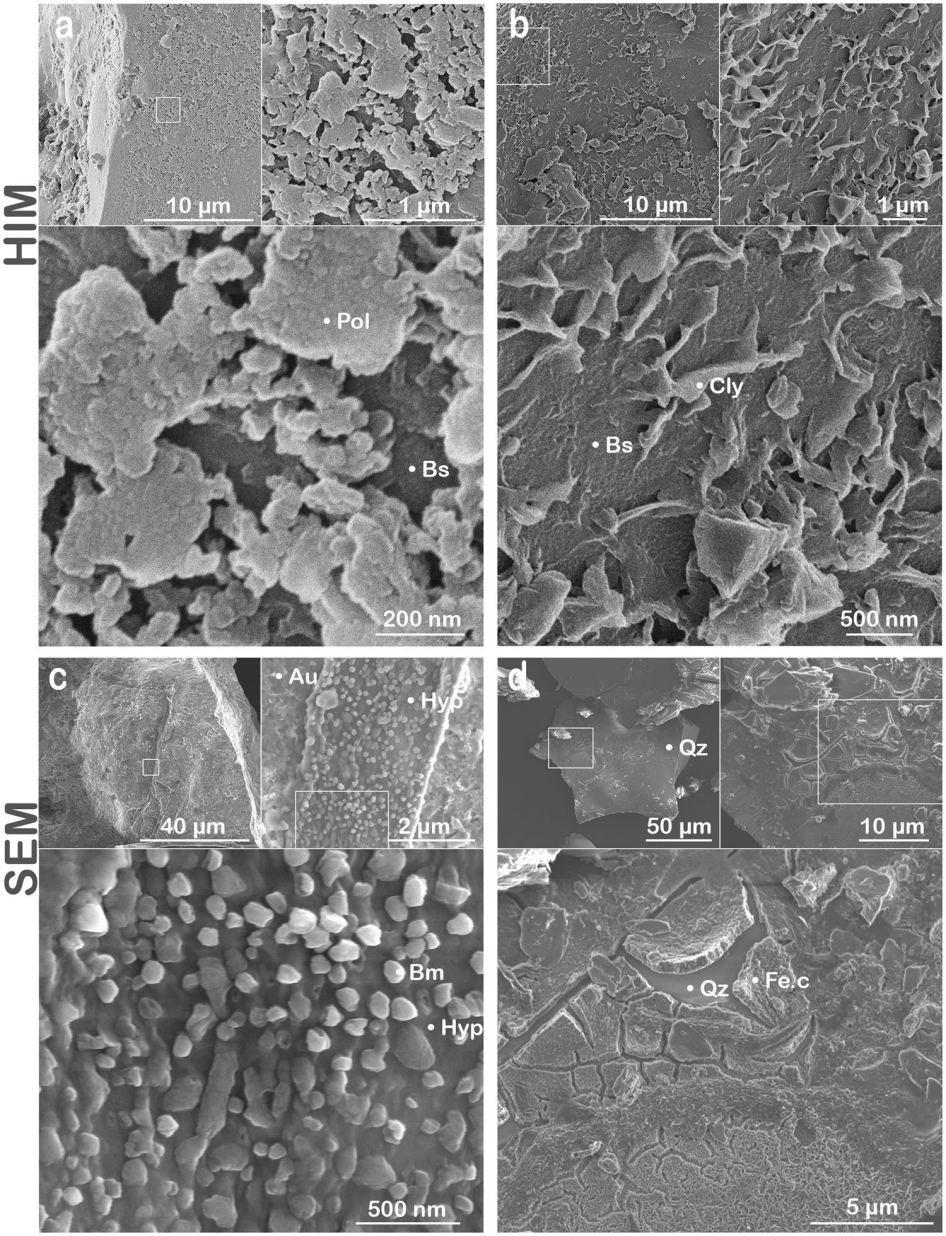
Mineral coatings. HIM and SEM micrographs showing grain coatings on mineral surfaces and secondary mineral formation for granular samples deployed in the humid (**a**), (**b**), and (**d**) and subhumid (**c**) forest sites. Basalt retrieved from the humid (Calhoun mixed hardwood-pine forest) site showed (**a**) coatings on the corner of a basaltic grain including the initial stages of biofilm development with morphological features that resemble extracellular polymers (Pol) when analyzed by HIM. (**b**) Putative inorganic features resembling clay minerals (Cly) were also observed in the same basaltic sample. (**c**) Secondary mineral formation was assessed for the subhumid (Catalina conifer forest) site. Fungal growth on an augite (Au) grain showed putative biomineral precipitates (Bm) embedded in the fungal hypha as <250 nm nodules (e.g., biologically derived Fe-rich aggregates or Ca/Mg oxalates). (**d**) A SEM micrograph of a quartz grain with a desiccated iron-rich (Fe.c) coating that was ~1 µm in thickness.

**Figure 5:**
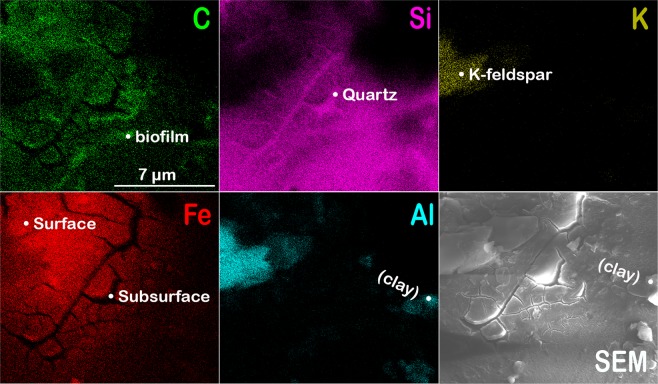
Elemental distribution on grain surfaces. Elemental maps captured by scanning electron microscopy for a quartz grain from a granite sample deployed in the humid (Calhoun hardwood forest) environment (Fig. 4d) that show the distribution of surface carbon and iron (mineral coating), subsurface silicon of underlying quartz, potassium and aluminum (likely from an adjacent feldspar) together with a SEM micrograph of the sample area.

In this study, we present imagery and data collected by HIM and SEM that provide potential evidence of (i**)** biomechanical rock disruption manifested by fungal-enhanced expansion of mineral sheets (Fig. 3) and (ii) biochemical transformation of rock demonstrated by the development of nanocoatings (Figs 4 and 5), the formation of secondary minerals (Fig. 4), and incipient weathering features observed at microbe-mineral interfaces (Fig. 2). Biomechanical disruption is a form of weathering that involves fungal hyphae and microbes penetrating minerals along crystal planes or tunneling into minerals^25^. The turgor pressure exhibited by hyphae permit fungi to expand and contract as they selectively acquire nutrients from various minerals^17,46^. Fungal features in our study exhibited contrasting growth patterns, sizes, and surface morphologies across sites that likely represent a variety of fungal species or hyphal structures growing in the different environments (Figs 2 and 3). For instance, fungi present smooth, flat hyphae (Fig. 2b) that grow along grain edges (Fig. 3b,d) or in between mineral sheets (Fig. 3c). Fungal structures were also cylindrical in shape (Fig. 3a) and displayed spheroids adhered to its surfaces that may indicate a microbial symbiont (Fig. 3a).

Biochemical weathering, often concurring with its mechanical counterpart, is mediated by microorganisms through mechanisms that include: the release of compounds that promote weathering, such as organic acids, chelators, and reactive species (e.g., H^+^). These compounds originate from the organisms themselves or as by-products from the microbial decomposition of organic matter. Organic acids oxidize to produce carbon dioxide that results in greater concentrations of carbonic acid in solution^47^. The resultant higher pCO_2_ levels in soil solutions are made even higher due to plant and microbial respiration, which then creates more acidic, enhanced weathering conditions. Thus indirectly, microbe-mineral interactions enhance aggregate stabilization in soil, which allows for more effective, undisturbed weathering activity by bacteria and fungi^17,48^. Biochemically-induced changes to mineral surfaces following these processes involve pitting, etching, and other microtopographic changes to grain surfaces, mineral displacement and/or replacement by the formation of secondary nano-minerals, or the complete dissolution of mineral grains^17,18,49^.

### Biomechanical disruption of mineral sheets

Fungi comprised a major component of the biota interacting with the granular substrates in all field sites (Fig. 3a–d) and showed strong evidence of biomechanical weathering (Fig. 3b–d). Biomechanical interactions were observed along the edges and surfaces of biotite sheets from granite placed in the subhumid forest environment (Catalina mixed conifer forest; Fig. 3b-c). Fungal hyphae can be seen interacting with mineral surfaces by penetrating between the interlayer spaces of biotite along grain edges (Fig. 3b) or growing between exposed surface sheets of biotite (Figs 3c and S4). Similar to the Catalina conifer forest, hyphae in the humid Calhoun hardwood-pine forest colonized all three rock types, with hyphae extending between mineral sheets, adhering to grain surfaces, and growing along the edges of grains (Fig. 3d).

We present potential evidence for contrasting mechanisms of biomechanical weathering versus biochemical weathering including interactions along mineral basal surfaces, side edges, and between mineral sheets. Our results and prior research suggest that distinct fungal species use varied strategies to mine nutrients that may place them in different ecosystem niches or that fungi exhibit contrasting responses to varying environmental conditions. For example, laboratory studies identified unique weathering signatures for individual fungal species grown under sterile conditions with phlogopite mica, where each species exhibited specific growth patterns (e.g., branching on mineral surface, penetration of interlayer sheets) and biochemical weathering signatures when interacting with the minerals^50^. Biomechanical forcing served as an early stage weathering mechanism in the surface transformation of biotite incubated with ectomycorrhizal fungi in a laboratory experiment for 3 months^29^. Here, the authors identified a coupled mechanical-chemical mechanism beginning with biomechanical weathering followed by the chemical alteration of the biotite lattice structure at the fungal-mineral interface. Nutrient abundance and accessibility were also found to influence fungal-mineral interactions in a^33^P radiotracer study where direct arbuscular mycorrhiza contact with biochar led to the uptake of six times more phosphorus into host plant roots compared to biochar minerals where mesh excluded direct fungal-mineral contacts^51^. The biomechanically enhanced dissolution of minerals may also be affected by differences in microbial access to moisture in addition to being dependent on the physical characteristics of mineral grains (e.g., particle size and shape) and the microbial taxa present in a particular weathering environment^50^.

### Coatings on mineral surfaces

Microscopy revealed nanoscale coatings on mineral surfaces in the humid Calhoun forest that represent potential sites for incipient weathering (Fig. 4), yet we observed fewer coatings in our survey of mineral grains from the drier Catalina desert and mixed conifer sites (Fig. 2). Coatings in several of the Calhoun samples may be abiotic (i.e., chemically induced by nearby redox conditions) or may be attributed to microbial biopolymers. The latter is suggested by the patchy coatings associated with preliminary exudation of extracellular polymeric substances (EPS)^52^ (Fig. 4a), which may comprise the desiccated carbon- and iron-rich coatings detected on a quartz grain (Figs 4d and 5).

Mineral coatings were most pronounced in the wetter environment (downslope landscapes of Calhoun mixed hardwood-pine forest; Fig. 4a,b,d), suggesting that the mineral coatings could be abiotic and translocated via groundwater or that an early stage of biofilm formation was potentially detected on the edges of basaltic grains (Fig. 4a). Here, we define biofilms as exopolymeric matrices that differ from the surrounding environment^53^. Biofilms developed on mineral surfaces from our study would likely influence the magnitude and pathways of weathering, yet the factors that regulate biofilm formation remain a topic of ongoing investigation^53–55^. Nanoscale examination of the putative biofilm surfaces in our study revealed morphological characteristics of EPS (Fig. 4a)^52^. Neoformed features, possibly inorganic, were also present in the granular samples, with a closer examination suggesting the precipitation of clays onto the grains (Fig. 4b).

**Figure 6:**
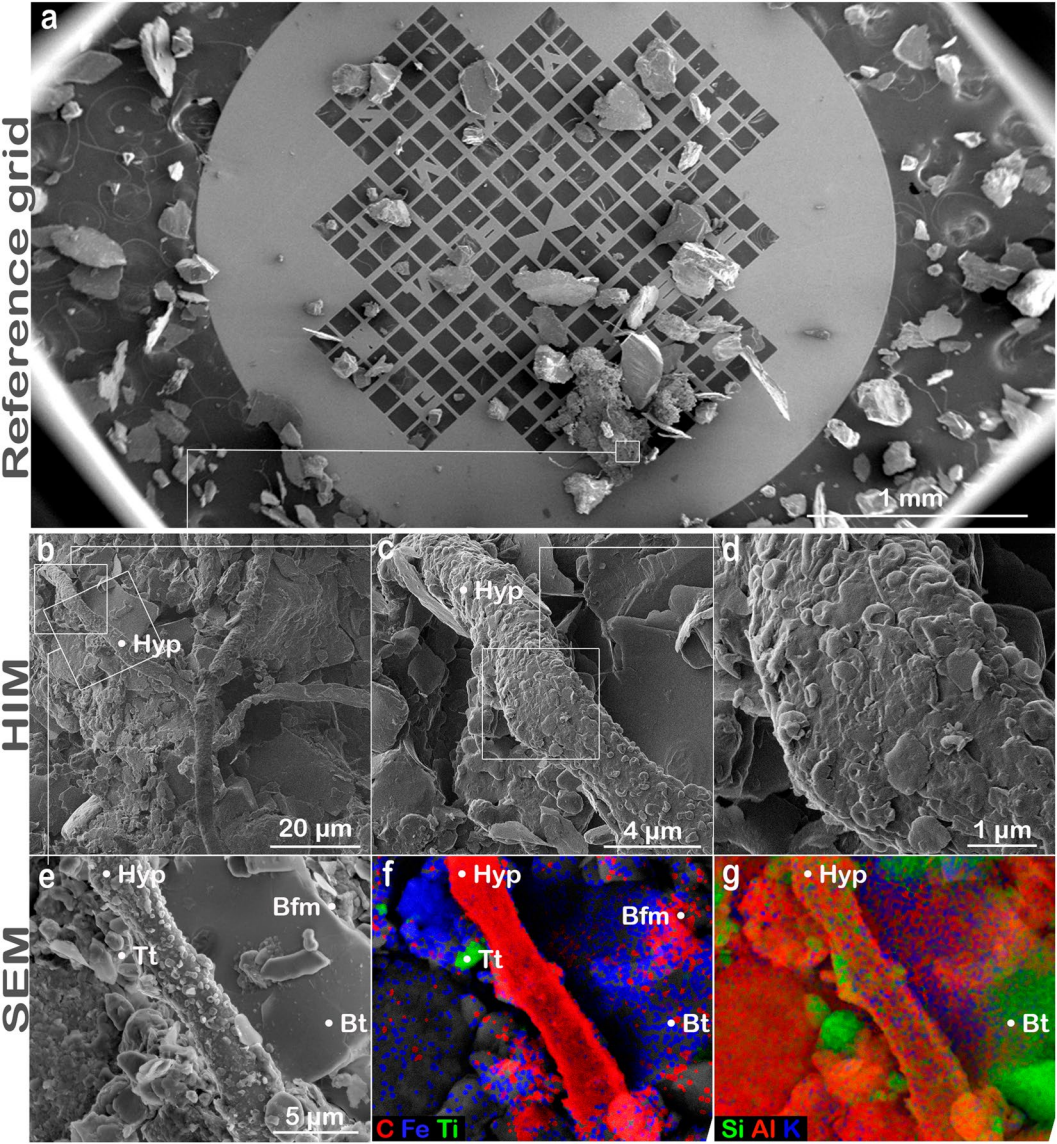
Demonstration of bimodal HIM-SEM co-location method. (**a**) Gilder reference locator grid used to image a fungal-mineral interface in a granite sample deployed in the humid environment (Calhoun forest) at the micro- to nanoscale using scanning electron microscopy (SEM; **a**,**e**) and helium ion microscopy (HIM; (**b**–**d**). (**f**) Elemental maps were generated for carbon (red), iron (blue), and titanium (green). The maps show carbon-rich fungal hypha (Hyp) and potential biopolymer films (Bfm) attached to the biotite (Bt) mineral grain. Fe-rich aggregates and Ti-rich (Tt) particles are also visible. Elemental maps were also generated for (**g**) silicon (green), aluminum (red), and potassium (blue).

An iron-rich coating was also identified by SEM on the edge of a quartz grain from granite buried in the humid Calhoun forest (Figs 4d, 5 and S6). The coating featured desiccation cracks that exposes the silicon-rich grain underneath (Figs 4d and S6). Imaging and element maps by SEM showed Fe and C in the coating that was distinct from the underlying quartz (Fig. 5). The iron-rich coatings may be abiotic or microbial in origin given their elevated carbon levels compared to the exposed quartz surface (Fig. 5). One hypothesis is that the coating is abiotic in origin where soluble iron from pore spaces in the highly weathered soil environment precipitated out of solution as a coating on the surface of the quartz. This highly reactive Fe-oxide coating may have scavenged carbon via adsorption or coprecipitation with the Fe oxide minerals^56^. Another hypothesis is that microbes enhanced the dissolution of biotite minerals in the granite substrate sample containing quartz minerals and that the coating we observed served as a pathway for microbes to retain dissolved Fe as an electron source. A laboratory study comparing biofilm formation on biotite to those on glass in Fe-deficient growth medium found that biofilms developed on biotite contained more biomass, higher specific numbers of viable cells, and greater base cation concentrations in the biofilm^53^. Micrographs from the aforementioned study also present evidence that biofilms weather biotite particles adhered to the grain and enhance the dissolution of reactive channels in the biotite surface, which is consistent with our imaging results that identified an Fe-rich aggregate associated with a fungal hypha that grew adjacent to a biotite grain (Fig. 6).

### Biomineral formation

Fungi promote soil aggregation and structural development by embedding grains into their hyphal network, and by forming secondary minerals that precipitate, in part, from the release of base cations (e.g., K^+^, Mg^2+^, Ca^2+^) during mineral weathering^23^. Biominerals formed by free-living and symbiotic fungi (e.g., mycorrhizae) include aggregated iron hydroxides, calcium and magnesium oxalates, among others^53,57^. We observed fungal hyphae adhered to an augite mineral in a basalt sample from the subhumid site (Catalina mixed conifer forest) that contained <250 nm aggregates embedded into a hyphal strand (Figs 4c and S5). We interpret this as potential evidence for biochemical weathering, specifically secondary biomineral formation (Fig. 4c). Based on our available chemical data (Fig. S5) and prior work^33^, we attribute the nanoparticulate secondary minerals attached to the hypha to fungal activity. We hypothesize that the aggregates may comprise Ca or Mg oxalates or Fe-rich precipitates secreted at the hypha surface. Point analyses detected Fe, Ca, and Mg in the energy dispersive spectra (Fig. S5); however, we were unable to identify the elemental composition of such biominerals due to the small size of the aggregates (SEM beam size limitations) and the proximity to augite.

Biominerals of comparable size (~1 µm) and morphology were documented during fungal diagenesis of carbonates, where Ca oxalate biominerals precipitated onto dolomite surfaces^27^. Similar to the secondary minerals on the hypha from our study (Fig. 4c), Cu, Cd, and Zn oxalates have been observed entrapped in hyphae, mycelial cords, and mucilaginous sheaths covering fungal biomass^58^. The accumulation of calcium oxalate on ectomycorrhizal hyphae can be mineral-specific and correspond linearly to the degree of calcium weathering from minerals as shown in a microcosm study for quartz, granite, basalt, and gabbro^59^. Oxalates are strong low molecular weight organic chelators of Al^3+^ and Fe^3+ ^^17^, which could be a fungal or plant-derived biochemical weathering agent in the Catalina mixed conifer forest. Another type of biomineral that may be produced by and adhere to fungal hyphae includes iron-rich aggregates, which have been recognized as <1 µm botryoidal minerals (globular external forms) produced during the fungal weathering of Icelandic basalt^60^. Our findings provide support for biomineralization in a field setting, where fungi produced secondary minerals utilizing cations present in pore solution or from incipient basalt weathering.

### Co-locating microbe-mineral interfaces using HIM and SEM

**Figure 7:**
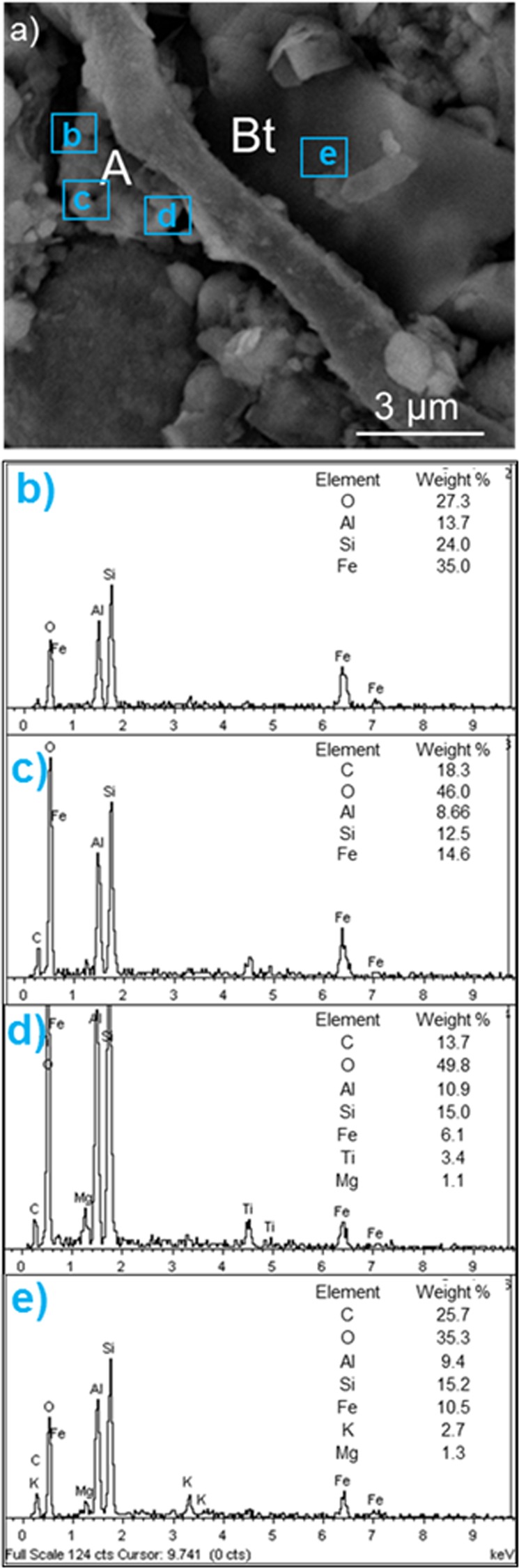
Energy dispersive X-ray spectra (EDX) for aggregated materials in a granite substrate sample from the humid Calhoun forest site. (**a**) Secondary electron images captured by SEM for the fungal-mineral interface presented in Fig. 6. (**b**–**d**) The composition of aggregated materials (abbreviated as A in image) were assessed using post-processing of compositional mapping and were comprised of silicon, aluminum, oxygen, carbon, iron, and titanium as indicated by EDX. (**e**) Biotite (Bt) was identified as the mineral associated with the fungal hypha.

We developed a method to overcome the limitation of HIM for quantifying elemental distribution and tested our technique by consecutively imaging the same microbial-mineral interface with both HIM and SEM (Fig. 6). A granular granite sample from the humid forest site (Calhoun mixed hardwood-pine forest) was mounted on a reference locator grid and imaged initially with SEM due to its large field of view (Fig. 6a). The sample was then surveyed using helium ion microscopy to identify both biological specimens that grew in the mesh bags during one year of deployment as well as potential biotic-abiotic weathering interfaces in the retrieved samples (Fig. 6b–d). The HIM micrographs revealed an association between a fungal hypha and mineral grain (Fig. 6b) and an aggregation of materials on the fungal hypha (Fig. 6c). Nanoscale imaging also showed bacterial cells that appeared to be attached to the hypha surface (Fig. 6d). Given that HIM provides no supporting elemental data, we revisited the same identified locations with SEM (displayed in Fig. 6e) to measure the chemical composition of the organic material and grain surfaces using elemental maps and point analyses (Fig. 6f,g). The aggregated particles, likely evidence of biomineral formation, were rich in iron, and appeared to contain titanium-rich particle(s) (Figs 6f and 7a–d). Energy dispersive spectra and mapped elemental distributions further showed that the hypha was associated with biotite (Figs 6f,g and 7e). Additional examples demonstrating the success of the bimodal HIM-SEM imaging method for analyzing mineral grains and potential weathering interfaces were documented (Figs S7, S8).

Helium ion microscopy has been successfully integrated with other technologies to enhance the strength of its superior imaging capabilities with additional quantitative measurements. X-ray diffraction and ^43^Ca nuclear magnetic resonance were combined with HIM to enhance the molecular and microscopic assessment of well-known organic matter reference materials (Suwannee River natural organic matter, fulvic acid, and humic acid)^61^. Helium ion microscopy was also coupled with atomic force microscopy, fluorescence microscopy, 3D structured illumination microscopy, and direct stochastic optical reconstruction microscopy to characterize membrane nanodomains in mammalian cells, where HIM helped identify specimens with 1.5 nm resolution^44^. Our findings provide examples of microbe-mineral interfaces identified using HIM in field samples that would be well-suited for integration with other quantitative analyses. We envision combining our SEM-HIM technique with focused ion beam sectioning and transmission electron microscopy to quantify the geochemical composition of biomineral formation (Fig. 4c), coatings on mineral surfaces (Figs 4a,b,d), and to potentially detect element loss at fungal-mineral interfaces (Figs 2 and 3). Furthermore, the SEM-HIM approach provides the resolution required to assess the structures of clay minerals required by microbes. For example, the frayed edges of weathered minerals release elements required for microbial growth (i.e., K^+^ in biotite) from the mineral structure during the initial stages of weathering. Conversely, frayed edge weathering sites also represent nutrient refugia where the reduction of ferric octahedral clay minerals under low pore water oxygen concentrations provide sites that fix biologically relevant interlayer cations (e.g., K^+^, H_3_O^+^, and NH_4_^+^)^23^. Our coupled SEM-HIM method demonstrates a pathway to capture HIM micrographs of microbe-mineral interfaces at sub-nanometer resolution in combination with measures of elemental distribution by SEM. Our approach also shows strong potential for integration with additional microscopy and spectroscopy techniques.

## Conclusions

Microbes support terrestrial ecosystems by extracting and transferring rock-derived nutrients through direct and indirect weathering mechanisms. Our study is the first to illustrate how helium ion microscopy, in combination with scanning electron microscopy, can be successfully applied in the earth and ecosystem sciences to image the complex nanoscale environment of microbe-rock interfaces that are keystone in understanding incipient biogeochemical cycles. We demonstrate the effectiveness of a bimodal microscopy approach to assess how microbes interact with basalt, granite, and quartz deployed in semiarid, subhumid, and humid climates for one year. The method we employed identified the intricate morphology of exposed mineral surfaces prior to and following exposure to biological or chemical effects in the field. We visualized individual mineral grains and associated mineral-organic matter interactions at the micro- to nanoscale using helium ion microscopy. We then quantified the elemental distribution at the same referenced interface using point analyses and elemental maps produced by scanning electron microscopy. The results enhance our understanding of incipient mineral weathering in complex field systems by characterizing the biomechanical expansion of mineral sheets by soil fungi in all three climates at sub-nanometer resolutions. Rock substrates deployed in the humid mixed hardwood-pine environment also present evidence for putative biomineral formation and the development of coatings on mineral surfaces that represent early sites of biochemical transformation. Coupling the superior imaging capabilities of HIM with the ability to investigate elemental composition by SEM helped address how microbes interact with mineral surfaces in natural settings by assessing nanoscale features in combination with measures of geochemistry on mineral surfaces.

## Methods

We addressed our research objective of assessing potential contributors to mineral weathering in field systems by utilizing in-soil mesh bags filled with unreacted basalt, granite, and quartz sand deployed in three climates for one year. Samples were buried in semiarid and subhumid landscapes in the Catalina Critical Zone Observatory (CZO) and humid environments in the Calhoun CZO. All samples were processed at Oregon State University and analyses were conducted with the Environmental Molecular Sciences Laboratory at the Pacific Northwest National Laboratory.

### Field setting

Sites extend from water-limited systems in the southwest US to wet, humid forests in the southeast. The Catalina CZO (Arizona) encompasses a semiarid to subhumid environmental gradient that spans significant range in average temperature (24–10 °C) and precipitation (25–85 cm) with well-defined vegetative communities^62,63^. Climate end members include desert scrub (1100 m.a.s.l.), characterized by Saguaro (*Carnegeia gigantea*), Acacia (*Acacia* spp.), Agave (*Agave* spp.), and Ocotillo (*Fouquieria splendens*); and conifer forest (2400 m.a.s.l.) including Douglas fir, Ponderosa Pine, and white fir. The Catalina CZO spans intrusive Precambrian to Tertiary parent rock^64,65^ with granitic intrusions and granitic soils that present a relatively narrow range in mineral composition and grain size^66,67^. Desert scrub soils formed on the Catalina granitic pluton (Oligocene–Miocene) and the mixed conifer soils on the Wilderness granite suite (two-mica granite; Eocene). The most common soil types in the Catalina CZO include Entisols (e.g., Torriorthents) in the desert scrub landscape as well as Mollisols and Entisols (e.g., Haplustolls, Ustorthents) in the mixed conifer forest^67^. The Calhoun CZO (South Carolina) represents the wet, humid end member of the considered climate gradient, receiving 127 cm of precipitation a year with an average annual temperature of 16 °C. Hardwood tree species comprise Shortleaf Pine (*Pinus echinata*), Northern Red Oak (*Quercus rubra*), and Sweetgum (*Liquidambar styraciflua*). The Calhoun sites are located within the Holcomb’s Branch watershed of the USFS Calhoun Experimental Forest where soils formed on gneiss bedrock (biotite-quartz-feldspar mineralogy) in the region of the Whitmire Complex or metadiorite in the Wildcat Complex comprised of plutons and sub-volcanics^68^. The most common soil orders classified for the watershed include Alfisols (e.g., Hapludalfs) and Ultisols (e.g., Kanhapludults)^69^. Herein, we reference three field areas by location and vegetation type: Catalina desert scrub, Catalina mixed conifer forest, and Calhoun mixed hardwood-pine forest.

### Experimental design

The in-soil mesh bag approach comprised granular granite, basalt, and quartz (53–250 μm) heat sealed into nylon mesh bags that (**i)** are reachable by fungal hyphae, referred to as “fungal-accessible” mesh bags (35 µm) and (**ii)** those that exclude fungi, or “fungal-excluding” mesh bags (0.5 µm). Our approach was adapted from established in-soil mesh bag methods that found the 35 µm mesh size excluded plant root growth into the bags while permitting the penetration of fungal hyphae^70,71^. We selected grain sizes that would enhance fungal foraging in the field based on work that showed ectomycorrhizal fungi displayed greater foraging activity in finer grain treatments (53–90 µm) compared to coarser granular substrates (500–1000 µm)^39^.

Mesh bags were autoclaved and buried for one year at 10 cm depth in three divergent-convergent landscape position pairs within each of the three field areas. A set of control mesh bag samples were also autoclaved, treated under identical conditions to the field-deployed samples, and stored at room temperature in the laboratory for the duration of the experiment. The mesh bags deployed in the field were distributed across three 1 m^2^ plots within each position to account for changes in microtopography. Upon retrieval, the mesh bags were preserved on dry ice to diminish microbial metabolism and stored at −80 °C prior to analysis on the HIM and SEM. The samples were not freeze-dried, air-dried or processed prior to analysis on the high resolution microscopes in order to minimize the formation of artifacts on the mineral surfaces.

### High resolution microscopy

Samples were prepared for the coupled SEM-HIM approach by utilizing a 200 mesh Cu Gilder reference locater grid (#G200F2, Ted Pella, Inc.) that was attached to an aluminum stub with double-sided carbon tape. A small subset of sample (~100 mg) was extracted from the mesh bag, sprinkled onto the grid, and gently pushed into the grid surface to secure the sample prior to loading the stub into the microscope. The samples were coated with a 10 nm carbon layer by thermal evaporation using a 108 C Auto Carbon Coater (Ted Pella, Inc.).

The SEM-EDX technique represents a semi-quantitative approach for identifying the elemental composition of minerals in granular substrates. Sample analyses were conducted with a FEI Helios NanoLab 600i field emission electron microscope. The Helios-EDX detector is an X-Max 80 mm^2^ Silicon Drift Detector (SDD) from Oxford Instruments. This detector is known for high stability and accuracy given its large analytical area and permits high resolution, high-count rate mapping at low and high energies. The SDD can detect lighter elements with accuracy starting from Be. The detector also performs high count rate mapping on rough surfaces and can identify phase composition for nanosize particles as small as 30 nm. The limitation in particle quantification thus is related to the electron beam penetration and excitation volume from the underlying substrate. The secondary electron images were collected at 5 kV voltage and 0.34 nA current at 4 mm working distance with the Everhart-Thornley detector (ETD), which is insensitive to takeoff collection angles. We collected both low and high magnification SEM images to find matching grain locations that were correlated with subsequent HIM micrographs. Briefly, we first located grains of interest using SEM and then located the identical grains for nanoscale imaging with HIM, and then revisited the same interface with SEM. Once HIM-SEM co-location was established for a site of interest, elemental mapping and point analyses were conducted to acquire relevant elemental data. The EDS analyses were performed at 10–20 kV voltage and 2–3 nA current using a backscattering TLD (through-the-lens) detector. Oxford INCA software was used to collect compositional maps and point spectrum analyses.

Sub-micron to nanoscale secondary electron images of the samples were obtained using an Orion Plus helium ion microscope (Carl Zeiss Microscopy, Peabody, MA). We used a 25 keV helium ion beam with probe current range of 0.1 to 1 pA to image the samples. Samples were transferred into the HIM via a load-lock system and were maintained at ~4 × 10^−7^ Torr vacuum during the imaging. When necessary, a low energy electron flood gun with beam energy of 500 eV was applied briefly interlaced with the helium ion beam so that charge build up on the surface was eliminated. Secondary electron images were collected using an Everhart-Thornley Detector. The image signal was acquired in line-averaging mode with a dwell time of 1 µs at a working distance of 7 to 8 mm. No post-processing procedures were applied to the digital images besides brightness and contrast adjustment.

We also used SEM and HIM to image unreacted control samples for the granular substrates that were sealed into mesh bags, sterilized with the field-deployed samples, and maintained in sealed containers at room temperature in the laboratory for the duration of the one-year field experiment. The control samples were imaged to confirm the absence of biological inputs in the initial substrate rock and to document the types of morphological surface features on the granular substrates at the start of the experiment (Figs 1 and S1–S3).

## Supplementary information

Supplementary Information

## Data Availability

All data generated or analyzed during this study are included in this published article (and its Supplementary Information files).
