# Supplementary material for: A coupled microscopy approach to assess the nano-landscape of weathering
Source: Sci Rep. 2019 Mar 29;9:5377. doi: 10.1038/s41598-019-41357-0 (PMC6441011; doi:10.1038/s41598-019-41357-0)
Supplement: Supplementary file 1 — Supplementary Information [file 41598_2019_41357_MOESM1_ESM.pdf]

Supplementary Information

A coupled microscopy approach to assess the nano-landscape of weathering

Rebecca A. Lybrand[a*], Jason C. Austin[b], Jennifer Fedenko[a], Rachel E. Gallery[c,d], Erin Rooney[a], Paul Schroeder[b], Dragos G. Zaharescu[e], and Odeta Qafoku[f]

[a]Department of Crop and Soil Science, Oregon State University, Corvallis, OR 97331, USA.
[b]Department of Geology, University of Georgia, Athens, GA 30602, USA.
[c]School of Natural Resources and the Environment. University of Arizona. Tucson, AZ, 85721, USA.
[d]Department of Ecology and Evolutionary Biology, University of Arizona. Tucson, AZ, 85721, USA.
[e]School of Earth and Atmospheric Sciences, Georgia Institute of Technology, Atlanta, GA 30332, USA.
[f]Pacific Northwest National Laboratory, Richland, WA 99352, USA.
*Corresponding author

Phone number: 541-737-1036; Fax number: 541-737-1589

Rebecca Lybrand e-mail contact: Rebecca.Lybrand@oregonstate.edu
Jason Austin e-mail contact: jayc.austin@gmail.com
Jennifer Fedenko e-mail contact: jennifer.fedenko@oregonstate.edu
Rachel E. Gallery e-mail contact: rgallery@email.arizona.edu
Erin Rooney e-mail contact: erin.rooney@oregonstate.edu
Paul Schroeder e-mail contact: schroe@uga.edu
Dragos Zaharescu e-mail contact: zaharescu@gatech.edu
Odeta Qafoku e-mail contact: Odeta.Qafoku@pnnl.gov

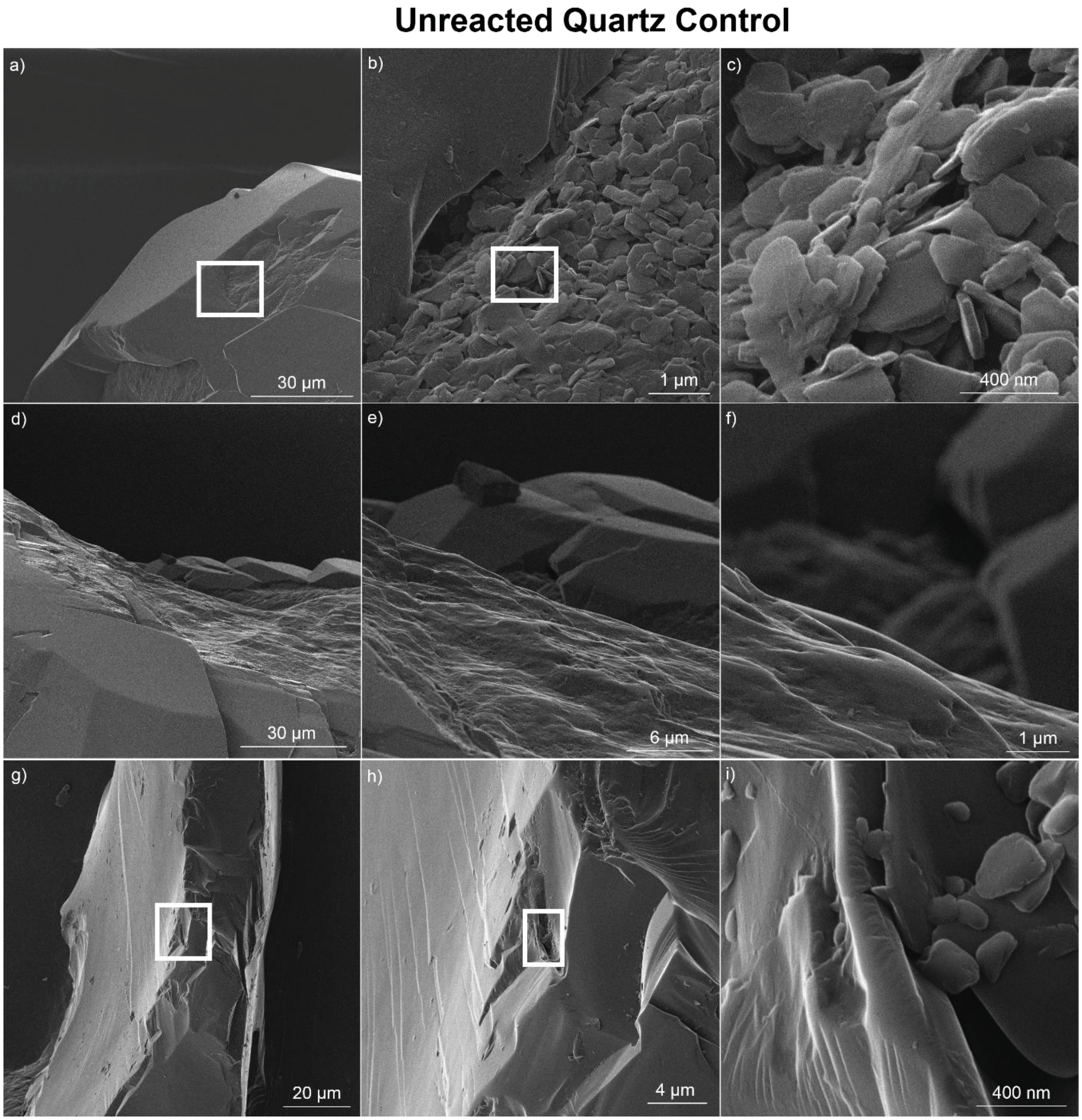

Figure S1. Helium ion micrographs of unreacted quartz grains. Undeployed samples were imaged for comparison with the field-deployed samples in the study.

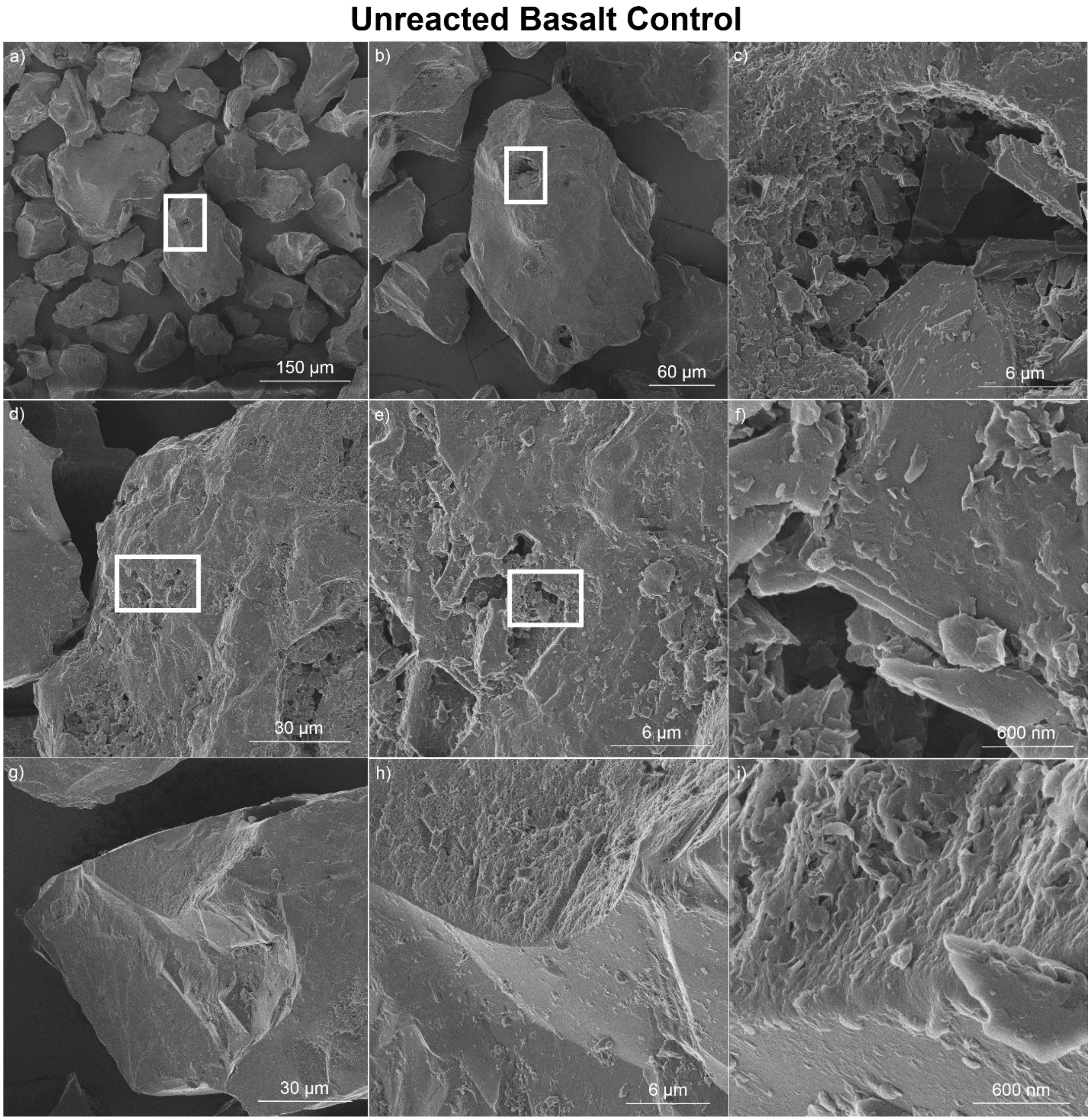

Figure S2. Helium ion micrographs of unreacted basalt grains. Undeployed samples were imaged for comparison with the field-deployed samples in the study.

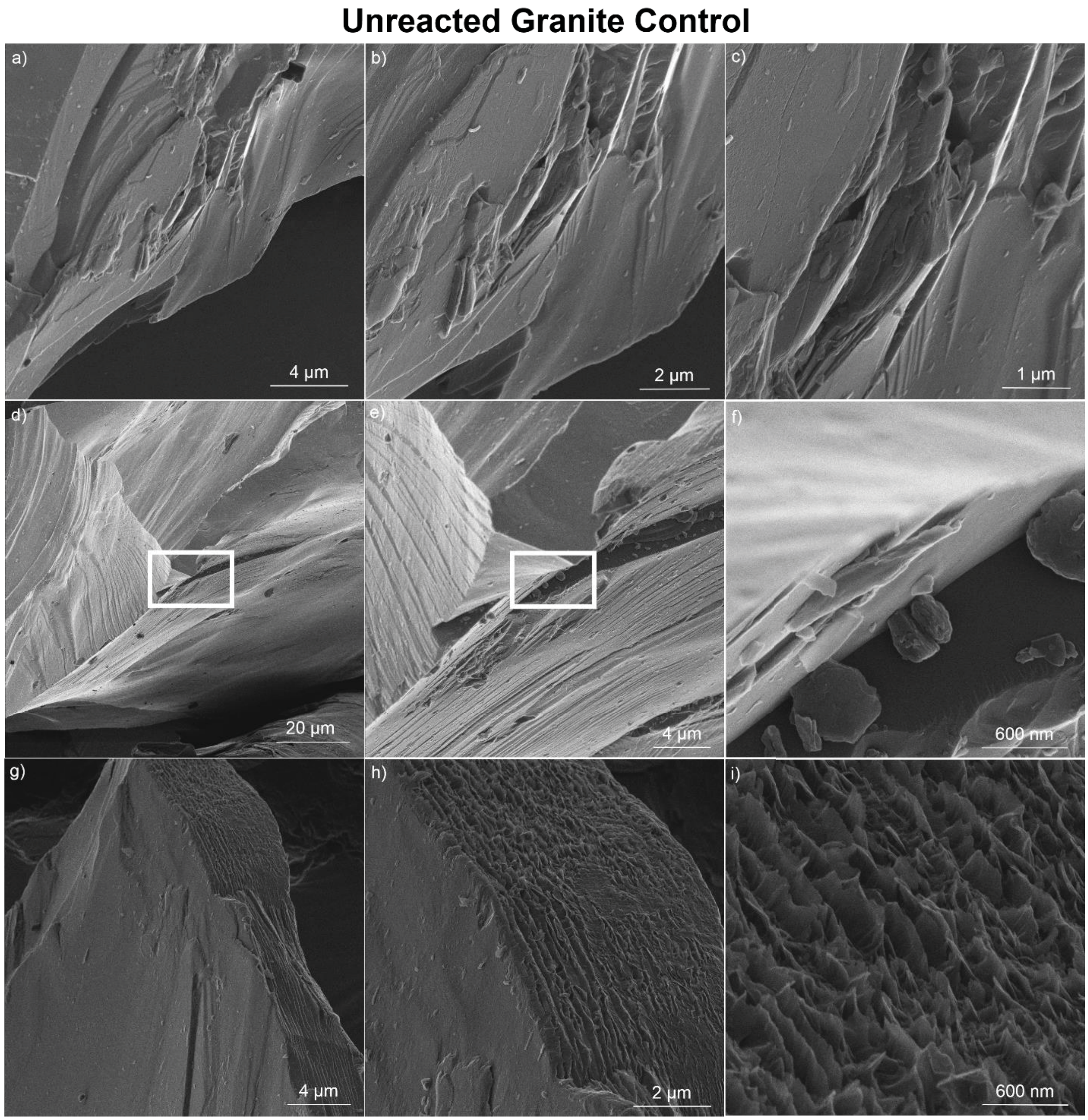

Figure S3. Helium ion micrographs of unreacted granite grains. Undeployed samples were imaged for comparison with the field-deployed samples in the study.

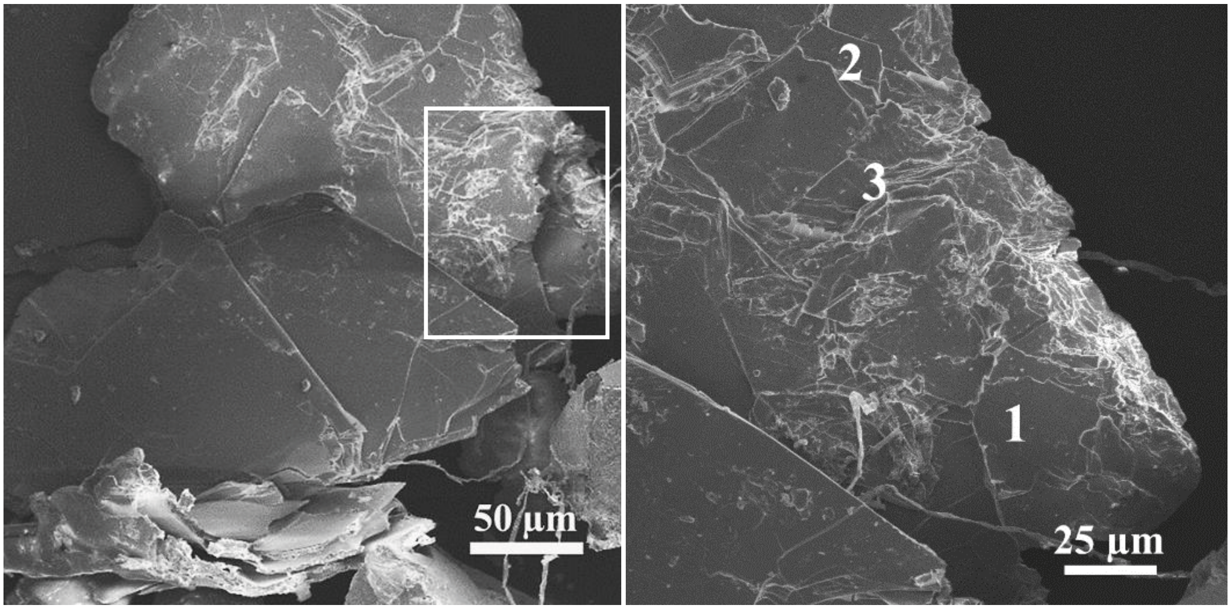

Identification of Biotite	C	O	F	Mg	Al	Si	Na	K	Mn	Ti	Fe
	weight %										
Point 1	3.27	39.36	3.05	6.36	7.88	17.51		7.79	0.45	0.96	13.37
Point 2	4.07	44.47	4.14	6.61	7.06	16.03		6.35	0.39	0.69	10.19
Point 3	4.45	40.30	3.13	6.42	7.11	16.69	0.15	7.38	0.51	0.89	12.99
Mean	4.26	41.38	3.44	6.46	7.35	16.74	0.15	7.17	0.45	0.85	12.18
Std. dev.	0.27	2.78	0.61	0.13	0.46	0.74	NA	0.74	0.06	0.14	1.74

Figure S4. Identification of biotite. Secondary electron images and table containing elemental data from EDS point analyses used to identify biotite mineral imaged in **Fig. 3c** using scanning electron microscopy.

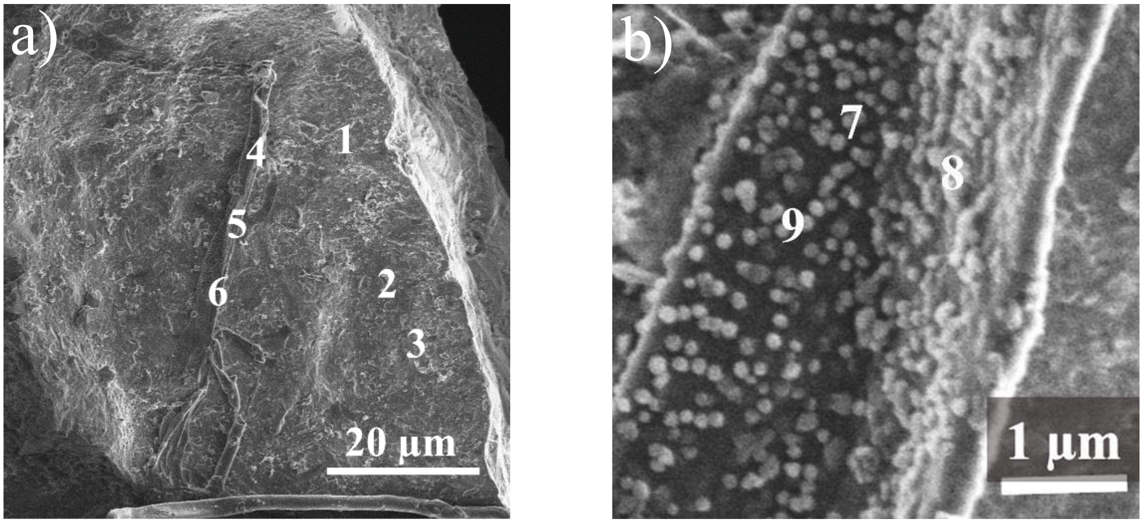

Mineral Surface (A)	C	O	Na	Mg	Al	Si	P	K	Ca	Ti	Fe
	----------	----------	----------	----------	----------	weight %	----------	----------	----------	----------	----------
Point 1	5.11	40.55	1.95	2.25	5.93	13.89		0.29	3.57	0.54	25.92
Point 2	6.72	40.47	1.76	3.65	3.91	11.80	0.22	0.49	3.83	0.75	26.41
Point 3	4.12	40.89	1.10	3.56	4.01	12.82	0.25	0.30	5.43	0.86	26.68
Mean	5.42	40.68	1.43	3.61	3.96	12.31	0.24	0.40	4.63	0.81	26.55
Std. dev.	1.84	0.22	0.45	0.78	1.14	1.05	0.02	0.11	1.01	0.16	0.39

Fungal Hypha adhered to mineral (A)	C	O	Na	Mg	Al	Si	P	K	Ca	Ti	Fe
	----------	----------	----------	----------	----------	weight %	----------	----------	----------	----------	----------
Point 4	31.51	35.72	0.77	1.26	5.38	10.08	0.20	0.46	2.36	0.31	11.93
Point 5	34.42	33.79	0.55	1.88	2.08	5.19	0.27	0.16	2.71	0.39	18.57
Point 6	29.69	33.24	2.11	2.41	2.75	10.22	0.33	0.30	4.12	0.93	13.90
Mean	32.06	34.25	1.14	1.85	3.40	8.50	0.27	0.31	3.06	0.54	14.80
Std. dev.	3.34	1.30	0.84	0.58	1.74	2.86	0.07	0.15	0.93	0.34	3.41

Magnification of fungal hypha with attached aggregates (B)	C	O	Mg	Al	Si	Ca	Fe
	----------	----------	weight %	----------	----------	----------	----------
Point 7	65.38	15.76	1.32	2.88	5.12		9.54
Point 8	46.50	18.82	2.72	4.26	9.46	2.32	15.93
Point 9	59.73	19.15	2.11	2.79	7.59		8.63
Mean	53.12	17.91	2.05	3.31	7.39	2.32	11.37
Std. dev.	9.36	1.87	0.70	0.82	2.18	NA	3.98

K emission line was used for all the elements except for Fe that L emission line was used.

Figure S5. Identification of augite. Secondary electron images and table containing elemental data from EDS point analyses used to identify augite imaged in **Fig. 4c** using scanning electron microscopy. Point analyses were also taken for aggregates attached to fungal hypha; however, we were unable to identify the exact chemical composition of the aggregates given their small size (<500 nm) and beam size limitations on the SEM. The accelerating voltages for the data presented in this figure included 10kV for points 1 to 6 and 5KV for points 7 to 9.

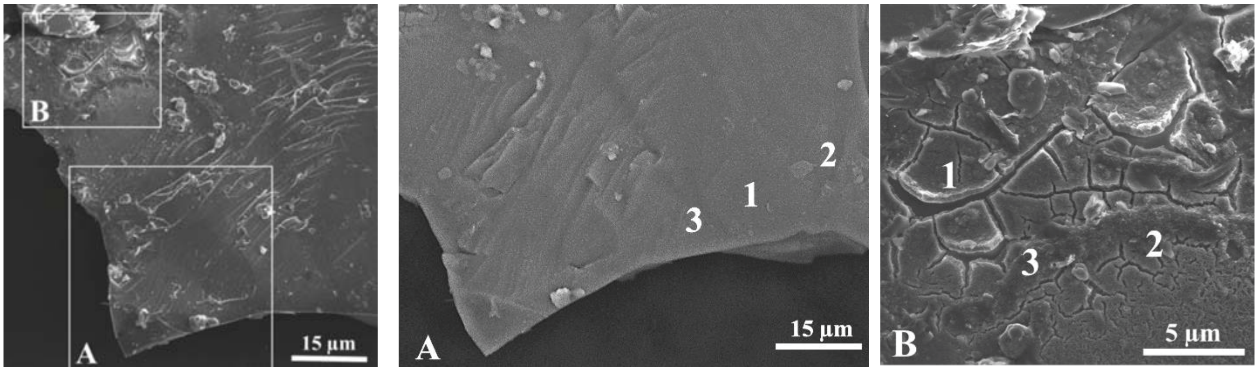

Identification of Quartz grain (A)	C	O	Si
	---------Weight %---------		
Point 1	16.80	39.56	43.64
Point 2	5.29	53.61	41.09
Point 3	14.88	44.27	40.85
Mean	12.32	45.81	41.86
Std. dev.	6.17	7.15	1.55

Coating on Quartz surface (B)	C	O	Si	Al	Fe	P	Ti
		----------------------Weight %-------------------					
Point 4	15.56	50.09	29.10	0.33	4.92		
Point 5	16.83	50.09	28.40	0.82	3.67	0.19	
Point 6	12.94	50.02	32.16	0.94	3.85		0.09
Mean	15.11	50.07	29.89	0.70	4.15	0.19	0.09
Std. dev.	1.98	0.04	2.00	0.32	0.68	NA	NA

Figure S6. Identification of quartz grain and chemical composition of coating on quartz surface. Secondary electron images and table containing elemental data from EDS point analyses used to identify elemental composition of the quartz grain and coating on quartz surface that are shown in **Fig. 4d** and **Fig. 5**.

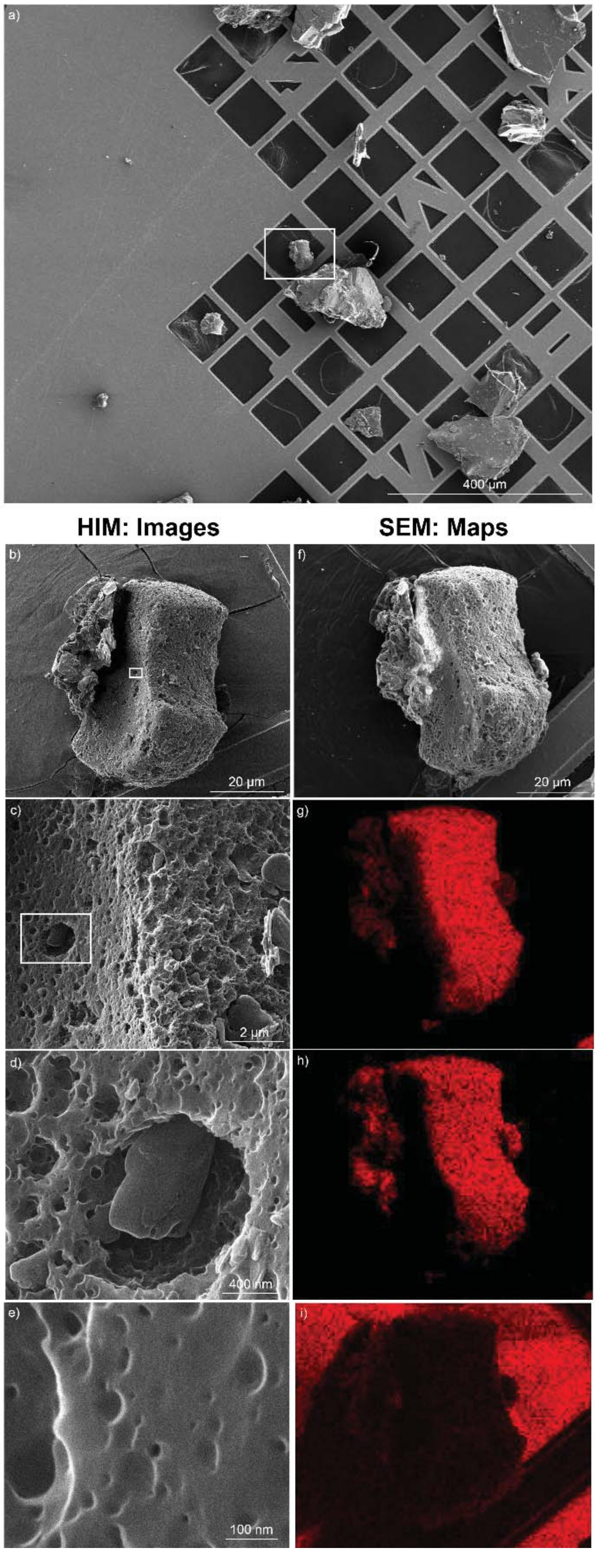

Figure S7. Demonstration of coupled HIM-SEM approach. a) Gilder reference locator grid used to identify and analyze minerals using scanning electron microscopy. **b-e)** Micro- to nanoscale micrographs captured for a mineral using helium ion microscopy. **f)** Secondary electron image for the same individual mineral using scanning electron microscopy. Elemental maps of the mineral were also generated for **g)** silicon, **h)** oxygen, and **i)** carbon.

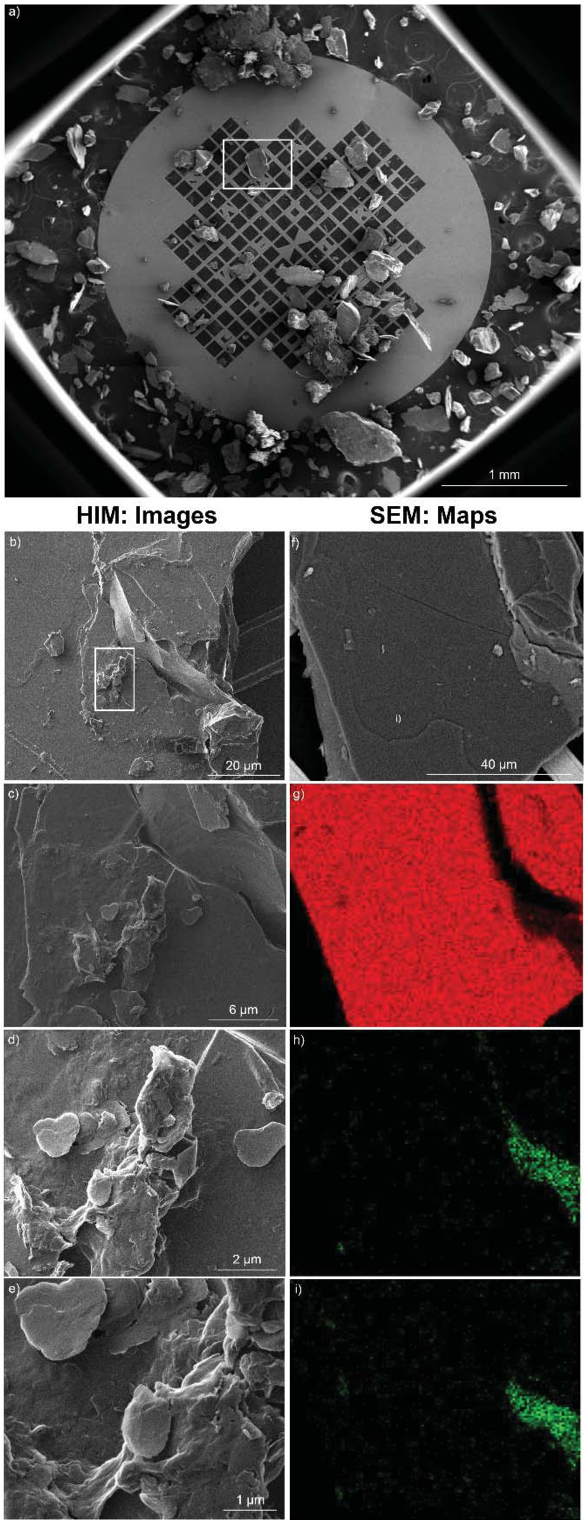

Figure S8. Demonstration of coupled HIM-SEM approach. a) Gilder reference locator grid used to identify and analyze minerals using scanning electron microscopy. **b-e)** Micro- to nanoscale micrographs captured for a mineral surface using helium ion microscopy. **f)** Secondary electron image for the same individual mineral using scanning electron microscopy. Elemental maps of the mineral were also generated for **g)** silicon, **h)** iron, and **i)** potassium.
